# Genetic Insights into the Giant Keyhole Limpet (*Megathura crenulata*), an Eastern Pacific Coastal Endemic: Complete Mitogenome, Phylogenetics, Phylogeography, and Historical Demography

**DOI:** 10.3390/genes15101303

**Published:** 2024-10-08

**Authors:** Brenda Bonett-Calzada, Fausto Valenzuela-Quiñonez, Miguel A. Del Río-Portilla, Natalia J. Bayona-Vásquez, Carmen E. Vargas-Peralta, John R. Hyde, Fabiola Lafarga-De la Cruz

**Affiliations:** 1 Centro de Investigacion Científica y de Educacion Superior de Ensenada (CICESE), Ensenada 22860, Baja California, Mexico; bonett@cicese.edu.mx (B.B.-C.); cevargas@cicese.edu.mx (C.E.V.-P.); 2 Departamento de Ecología Pesquera, Centro de Investigaciones Biológicas del Noroeste S.C., La Paz 23205, Baja California Sur, Mexico; fvalenzuela@cibnor.mx; 3 Division of Natural Science and Mathematics, Oxford College of Emory University, Oxford, GA 30054, USA; natalia.juliana.bayona.vasquez@emory.edu; 4 NOAA Fisheries Southwest Fisheries Science Center, La Jolla, CA 8901, USA; john.hyde@noaa.gov

**Keywords:** Fissurella, mitogenome, genetic diversity, population structure

## Abstract

Background: The giant keyhole limpet *Megathura crenulata* is a gastropod mollusk (Fissurella superfamily) that is endemic to the eastern Pacific coast from southern California, USA, to Baja California Sur, Mexico. *M. crenulata* is socioeconomically important as it produces a potent immune-stimulating protein, called Keyhole Limpet Hemocyanin, which is extracted in vivo and utilized for vaccine development. However, ecological studies are scarce and genetic knowledge of the species needs to be improved. Our objectives were to assemble and annotate the mitogenome of *M. crenulata*, and to assess its phylogenetic relationships with other marine gastropods and to evaluate its population genetic diversity and structure. Methods: Samples were collected for mitogenome assembly (*n* = 3) spanning its geographic range, Puerto Canoas (PCA) and Punta Eugenia (PEU), Mexico, and California (CAL), USA. Total DNA was extracted from gills sequenced using Illumina paired-end 150-bp-read sequencing. Reads were cleaned, trimmed, assembled *de novo*, and annotated. In addition, 125 samples from eight locations were analyzed for genetic diversity and structure analysis at the *16s rRNA* and *COX1* genes. Results: The *M. crenulata* mitogenomes had lengths of 16,788 bp (PCA) and 16,787 bp (PEU) and were composed of 13 protein-coding regions, 22 tRNAs, two rRNAs, and the D-Loop region. In terms of phylogeographic diversity and structure, we found a panmictic population that has experienced recent demographic expansion with low nucleotide diversity (0.002), high haplotypic diversity (0.915), and low *φ_ST_* (0.047). Conclusions: Genetic insights into the giant keyhole limpet provides tools for its management and conservation by delimiting fishing regions with low genetic diversity and/or genetically discrete units.

## 1. Introduction

The giant keyhole limpet, *Megathura crenulata*, is a gastropod mollusk of the Fissure-llidae family that inhabits rocky substrates along the Eastern Pacific Ocean coastal zone. Its distribution begins in Point Conception, California, USA, and extends south to Isla Asunción, Baja California Sur, México [1,2]. It is important socioeconomically as a high nutritional resource for human consumption, as limpets are an excellent source of proteins, vitamins (A, D), and other elements such as phosphorus and iron [3,4]. It also has high biomedical value in the pharmaceutical industry [5] as it produces a potent immune-stimulating protein called Keyhole Limpet Hemocyanin (KLH). This protein cannot be synthesized *de novo*, thus requiring its extraction from living organisms [6]. In recent years, KLH has been used as a carrier protein to generate hapten antibodies and peptides, demonstrating an expanding application in the development of experimental vaccines against pathogens [7]. Therefore, the KLH protein significant potential for use across biomedical disciplines [6,7,8,9].

In the United States, *M. crenulata* fishery is mainly regulated for the extraction of the KHL protein, human consumption is insignificant. In Mexico, it is not currently the target of commercial fisheries, but it has been used for human consumption. Since the 1990s, this limpet has been harvested sporadically by artisanal fisheries, with the highest production recorded of 180 tons in 1994. After that, its production decreased to 50 tons per year in 2001–2002 and an average of six tons per year during 2003–2015 [10]. The reduction observed in 2013, similar to that of other valuable benthic invertebrates, was associated with warming events in 2012 [11]. This resource plays an essential ecological role as a foundation species and key grazer in the temperate kelp forest ecosystems of the peninsula of Baja California [2,10]. There is a need for more information regarding this resource, as it has the potential to support a managed fishery and aquaculture industry in the future due to its nutritional and biomedical value.

Research on the giant keyhole limpet has mainly focused on its biological characteristics, such as natural distribution [2], growth [5], and trophic ecology [12]. However, there is still a lack of information on its phylogeography. It is crucial to understand the genetic diversity and differentiation of *M. crenulata* individuals across its distributional range. This knowledge would assist those interested in the resource to understand gene flow, barriers to dispersal, and effective population sizes in its natural environment. This is especially important before considering its extraction and helps guide fisheries management and potential use in aquaculture.

High-throughput DNA sequencing techniques have significantly improved the analysis of wild populations, especially in cases where information regarding their genomic status is limited or absent. This has led to increased interest in studying the mitochondrial genome, which is characterized by its high copy number in cells, conserved gene content, variable gene order, high mutation rate, predominantly maternal inheritance, and, in some cases for gastropods, doubly uniparental inheritance (DUI) with minimal recombination [13]. These traits make it a valuable tool for several analyses to reconstruct taxonomic relationships between species [14] and to address phylogeographic analyses within species [15]. In marine gastropods, complete mitogenomes and mitochondrial genes, such as *16S rRNA* and cytochrome oxidase subunit I (*COX1*), have been used to reconstruct phylogenetic relationships in various species such as *Haliotis* spp., *Diodora graeca* and *Notocrater youngi* [16,17,18]. However, the genus *Megathura* has not been included in any phylogenetic or phylogeographic study to date.

Generating genomic information for non-model species enables the development of essential data resources for guiding optimal resource management, particularly for species with high economic potential, such as *M. crenulata*. Across its distributional range, there is a lack of fundamental knowledge about this species. Therefore, this research aims to describe the mitogenome of *M. crenulata*, compare it with other marine gastropods, and evaluate the diversity and genetic structure associated with the *16S rRNA* and *COX1*.

## 2. Materials and Methods

### 2.1. Sampling for Mitogenome Analysis

The mitochondrial genomes of *M. crenulata* were assembled using total DNA extracted from three specimens collected at Puerto Canoas (PCA) and Punta Eugenia (PEU), Baja California, Mexico, and Channel Islands (CAL), California, USA. For each sample, gill tissue used for DNA extraction. The Phenol-Chloroform-Isoamyl Alcohol protocol [19] was used for DNA extraction from the PCA sample, and the commercial KingFisher™ Cell and Tissue DNA kit by Thermo Fisher Scientific (Waltham, MA, USA), was used for the PEU and CAL samples, following the supplier’s specifications. Genomic DNA for each sample was sheared by sonication with a Bioruptor^®^, by Diagenode (Liege, Belgium) using two rounds of five cycles of 30 s of sonication and 30 s without sonication on the high setting. Library prep protocol was followed using the Kapa Biosystems^®^ Hyper Prep Kit (KR0961–v4.15) by Roche (Basel, Switzerland), ligating custom adapters stubs and amplification through 12 cycles of PCR with custom nucleotide indexed primers [20]. A dual-size selection with magnetic SpeedBeads by Thermo-Scientific, Waltham, MA, USA [21] was performed to recover fragment sizes of ~250–450 bp. The resulting library from the PCA sample was sequenced using an Illumina HiSeq 4000, by Ilimuna, Inc. (San Diego, CA, USA), generating paired-end 150 nucleotide reads at the Oklahoma Medical Research Foundation Clinical Genomics Center; the PEU and CAL libraries were sent to Omega Biotechnologies for sequencing.

### 2.2. Bioinformatic Analysis

The PEU sample sequence quality analysis was performed using the QIAGEN CLC Genomics Workbench v10 (CLC; https://digitalinsights.qiagen.com; accessed in 1 January 2022) software, whereas the PCA sample quality analysis was conducted using both CLC and Geneious v10 (https://www.geneious.com, accessed in 1 January 2022), wherein low-quality sequences (quality limit = 0.05) were removed. Subsequently, *de novo* assembly was performed with the following specific parameters: filtering to keep contigs with depth coverage greater than 1.5× and containing more than ten reads, and a *de novo* assembly was carried out with both programs (Geneious and CLC Genomics Workbench).

For the PCA sample, two contigs containing the *M. crenulata* mitogenome were obtained, Geneious produced a 16,788 bp contig (mcre_gen_3), while CLC Genomics Workbench produced a shorter contig of 15,155 bp (mcre_clc3). The difference in contig lengths is likely due to variations in the assembly algorithms between the two software programs. The resulting contigs were aligned to resolve these discrepancies, revealing two gaps (69 bp and 1566 bp) in the shorter contig (mcre_clc3). Consequently, to circularize and complete the contig mcre_clc3, two primers were designed to amplify the missing fragments by PCR.

The sequences from the CAL sample were of low quality, and the complete contig for the mitogenome of *M. crenulata* was not able to be retrieved. However, with the CLC Genomics Workbench, three small contigs were obtained and successfully aligned with the mitogenome generated from the PCA and PEU samples.

### 2.3. Primer Design to Circularize the Mitogenome

To circularize the mitogenome of *M. crenulata* from the PCA sample, two sets of specific primers were designed to amplify the two missing fragments from the assembly of PCA sample. The primers used to amplify a 1446 pb missing fragment were F:5′TGAGAGACCAGGATTAGATACCCT3′, and R:5′GGGGCATGTATTTGCCGAGT3′, beginning at position 3771 and ending at 5217. This missing fragment includes 575 bp of the *12s rRNA*, *tRNA-Val* and 802 bp of the *16s rRNA*. To amplify the larger missing fragment (1681 bp), the primers used were F:5′TTTATGCGAATGAGCACGCC3′ and R:5′TGAGAGCAGCCCCTTTCTTG 3′, starting at position 9724 and ending at 11,404. This amplified fragment includes 23 bp of the *ATP6* gene (at the beginning of the amplicon) and 1197 bp of the *ND5* gene end of the amplicon, respectively). Between these genes, we found the D-loop region, which spans 461 bp. To design this set of primers, the software primer3plus was used [22]. To amplify these regions, the following final concentrations were used in a volume of 25 μL: Kapa buffer A 1×, MgCl_2_, 1 mM, dNTPs 0.2 mM, primers 0.2 mM each, Taq polymerase Kapa 0.4U, DNA 50 ng. The annealing temperature was 57 °C and 58 °C for each fragment, respectively, with 25 cycles. The PCR products were visualized by electrophoresis using 1% agarose gel and bidirectional Sanger sequencing was conducted by Eton Bioscience Inc (San Diego, CA, USA). A consensus mitogenome contig was built using the data from these results.

### 2.4. Mitogenome Annotation

The contigs of PCA and PEU were utilized for gene annotation via the Mitos web server [23], applying default settings tailored for invertebrate mitogenomes. Additionally, protein translation verification was carried out using the ExPASy translate tool (https://web.expasy.org/translate/; accessed in 1 August 2023). The tRNAscan v2.0 webserver was used to find and validate the tRNAs. However, this software did not identify arginine (*tRNA-Arg*), proline (*tRNA-Pro*), asparagine (*tRNA-Ans*), and serine-I (*tRNA-Ser-I*) tRNAs; these were annotated through comparison with the mitochondrial genomes of *Diodora graeca* and *Fissurella volcano* (GenBank accession numbers in Table 1).

### 2.5. Phylogenetic Analyses of Marine Gastropods

Phylogenetic analysis used the complete mitogenome sequences of *M. crenulata* from PCA and PEU, which were aligned with the complete mitogenome sequences from seven other species of the Lepetellida order of marine gastropod mollusks (Table 1). The alignment and subsequent analyses were conducted using QIAGEN CLC Genomics Workbench, v20 (https://digitalinsights.qiagen.com; accessed in 1 January 2023). Default settings in the jModelTest2 program [29] were employed to determine the best-fitting nucleotide substitution model based on the Akaike information criterion (AIC) and Bayesian information criterion (BIC). The selected model was the general time reversible evolution (GTR) combined with a γ distribution system model, incorporating a γ-distributed rate variation between sites (GTR + G + I), and implemented using a Markov Chain Monte Carlo simulation. Phylogenetic trees were generated over 20,000 generations, one tree was sampled every 100 generations, and default settings were inferred using the GTR+G+I model in the MrBayes 3.2 program [30]. Additionally, sequences of three protein-coding genes (*ATP8*, *ND6*, and *COX3*) previously identified in the CAL sample were extracted from the eight gastropod species in Table 1. This reduced data set of sequences was subjected to the same phylogenetic analysis as the complete mitochondrial sequences. The model selected for this analysis was GTR, combined with a γ distribution and incorporating GTR + G + I.

### 2.6. Sampling for Mitochondrial Genetic Diversity and Structure Analysis

The analysis of *16S rRNA* and *COX1* fragments included 125 samples collected from locations spanning nearly the entire species distributional range: Channel Islands, California (CAL, *n* = 19), Ensenada (ENS, *n* = 25), San Quintín (STQ, *n* = 16), Isla San Jerónimo (SJO, *n* = 14), Puerto Canoas (PCA, *n* = 15), Punta Eugenia (PEU, *n* = 21), Bahía Asunción (BAS, *n* = 8), and Isla Guadalupe (IGP, *n* = 7). Total DNA was extracted from gill tissue using the Phenol-Chloroform-Isoamyl Alcohol protocol [18].

The *16S rRNA* gene fragments were amplified using gene-specific primers [31]. This gene was selected because it is highly conserved, allowing individual comparisons [16]. The primers for amplifying the *COX1* gene were designed from the mitochondrial sequence of *M. crenulata* generated in this study and created using the Primer3Plus software [22]. The *COX1* gene was chosen due to its higher mutation rate and suitability for resolving phylogenetic relationships among species [16]. Primers sequences for *COX1* amplification were F:5′TCTTGGGGACGGGGTTGA3′ and R:5′ACCATAGTGGCCGCTGTAAAA3′. To amplify *16S rRNA* and *COX1* regions, the following final concentrations were used in a volume of 25 μL: Kapa buffer A 1×, MgCl_2_ 1 mM, dNTPs 0.2 mM, primers 0.2 mM each, Taq polymerase Kapa 0.4U, DNA 50 ng. The annealing temperature was 55 °C and 56 °C, and the total number of cycles used were 30 and 28 for *16S rRNA* and *COX1*, respectively. PCR products were visualized by electrophoresis using 2% agarose gels, and the forward and reverse strands were Sanger sequenced at the Southwest Fisheries Science Center facility on an ABI 3730 (SWFSC, NOAA).

### 2.7. Diversity and Genetic Structure Analysis

Sequence quality, trimming, and alignment analyses were performed individually for *16S rRNA* and *COX1* genes. Then, sequences were concatenated and aligned using MEGA6 v.11 software [32]. Diversity parameters, including the number of haplotypes, haplotype richness, and nucleotide diversity, were assessed using DNASP version 6.12.03 [33]. In addition, genetic distances between the concatenated matrix of *16S rRNA* and *COX1* genes were calculated using the Jukes and Cantor model in Arlequin [34]. To assess the level of genetic structure, paired *ϕ_ST_* values between localities and their corresponding statistical probability values were calculated with Arlequin version 2.3 [34], and the *p*-value used after Bonferroni’s correction was 0.006 [35,36].

### 2.8. Historic Demographic Analyses

Demographic tests (*Tajima’s D*, *Fu’s F*, and *R*_2_) and mismatch distributions were performed, and significant values were tested with 10,000 replicates using DNASP version 6.12. 03 [33]. Mismatch distribution values were plotted using Sigmaplot v12 (Systat Software Inc., San Jose, CA, USA). HapStar v3 [37] and InkScape v1.3.2were used to construct a minimum spanning network to illustrate the distribution and relationship of haplotypes.

A Bayesian Skyline plot (BSP) analysis [38] was conducted to infer changes in the effective population size over time [39]. This analysis used only the *16S rRNA* and *COX1* concatenated matrix and haplotype collapsed data, where data redundancy is reduced and equal haplotypes are grouped, and *16S rRNA* and *COX1* matrix and was performed using BEAST v.2.7.6 software [40]. For this analysis, Markov chain Monte Carlo (MCMC) simulations were run for 25,000,000 generations, with parameter and tree sampling every 2500 generations. The first 10% of runs were discarded as burn-in. The convergence of the runs was verified using Tracer v.1.7.2, ensuring effective sample sizes (ESSs) exceeded 200. A prior clock rate of 0.0157 substitutions/site/million years was applied [41]; this substitution rate has been previously reported for marine invertebrates.

## 3. Results

### 3.1. Mitogenome Content and Organization

The *M. crenulata* assembly resulted in two complete mitogenome sequences from samples collected at PCA and PEU locations. Specifically, the PCA sample yielded 11,223,886 raw reads, while the PEU sample produced 7,883,934 raw reads, both leading to successful assembly of the full mitogenome. In contrast, only three contigs, corresponding to the *ATP8*, *ND6*, and *COX3,* genes were recovered from the third sample, collected at CAL, which generated 9,996,416 of raw reads but did not allow for the assembly of the complete mitogenome (see Section 3.2 Phylogenetic Analyses).

**Figure 1:**
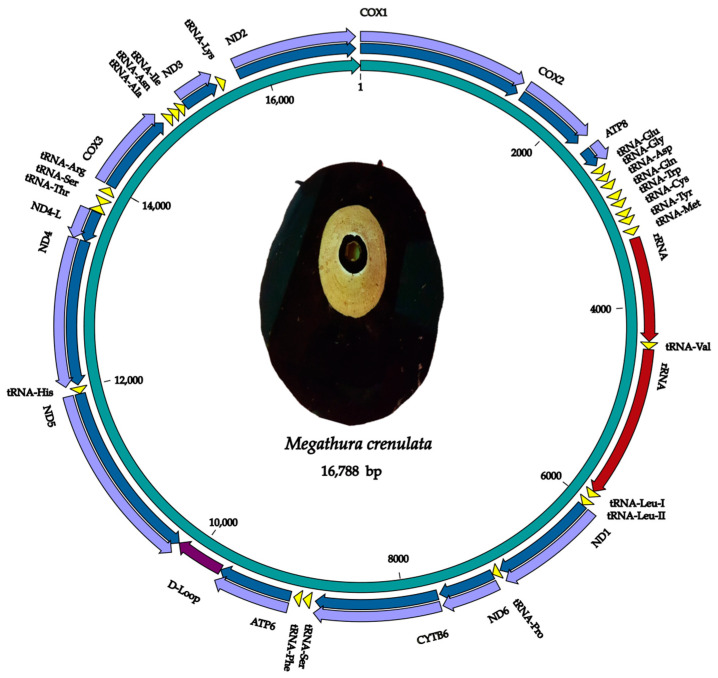
Map of the mitogenome of *M. crenulata* from the PCA sample. Arrows indicate the direction of transcription. Protein-coding genes (PCGs) are in purple, ribosomal RNA in red, transfer RNAs in yellow, D-Loop in dark purple, and the origin of replication in blue.

The consensus mitogenome is a double-stranded circular molecule of ≈16,788 bp (16,788 bp for the PCA sample and 16,787 bp for the PEU sample) with 54.4% AT and 45.5% GC content. It contains the usual 13 protein-coding genes (PCGs), two ribosomal RNA genes (rRNA), and 22 transfer RNA genes (tRNA) (Figure 1 and Table S1). However, four of these tRNAs (*tRNA-Pro*, *tRNA-Ser*, *tRNA-Arg*, and *tRNA-Asn*) matched in the MITOS server but not in the tRNAscan-SE web server. These sequences were annotated by similarity with the tRNAs of the mitochondrial sequences from *D. graeca* and *F. volcano*, generating possible secondary structures (Figure S1). In addition, two Leucine tRNAs (I and II) and two Serine tRNAs (I and II) are reported.

Of all these genes, 32 were on the heavy (+) strand, and the three PCGs *ND5*, *ND4*, *ND4-L*, and two transfer RNA genes (*tRNA-His* and *tRNA-Ser*) were coded on the light (−) strand.

Overall, PCGs constitute 63.7%, tRNAs comprise 14.7%, and ribosomal genes represent 9.5% of the mitogenomes of *M. crenulata*. The predominant initiation codon in PCGs is ATG, except for *ND5*, which utilizes ATA. The termination codon is predominantly TAA, except for the genes *ND6, CYTB*, and *ND3*, which are TAG. Additionally, the mitogenomes encompass 33 intergenic regions, covering a total of 1357 bp, and a region of 461 bp was detected between the *ATP6* and *ND5* PCGs, which we considered to be the control region (D-Loop) of the *M. crenulata* mitogenomes (Table S1).

The mitochondrial genomes of *M. crenulata* from the PCA and PEU samples were found to contain the same number of genes. However, when comparing the two mitogenomes, differences in nucleotide sequence were identified, mainly within the PCGs. These differences were identified in 16 genes, totaling 89 nucleotide substitutions (Table S2). The genes with the most significant differences were *COX1*, with 19 nucleotide substitutions, and *ND5*, with 13 nucleotide substitutions between samples (Table S2). However, in *COX1*, only 12 of these nucleotides resulted in amino acid changes, and in *ND5*, only one nucleotide led to an amino acid change, similar to what was observed in *ATP6* and *ND1* (Table S3). Specifically, amino acid changes in the *COX1* gene were observed at positions 424 to 436. In the *ND1*, *ATP6*, and *ND5* genes, amino acid changes were found at positions 6304, 9053, and 10227, respectively (Table S3), while all other nucleotide changes were synonymous substitutions.

### 3.2. Phylogenetic Analysis

**Figure 2:**
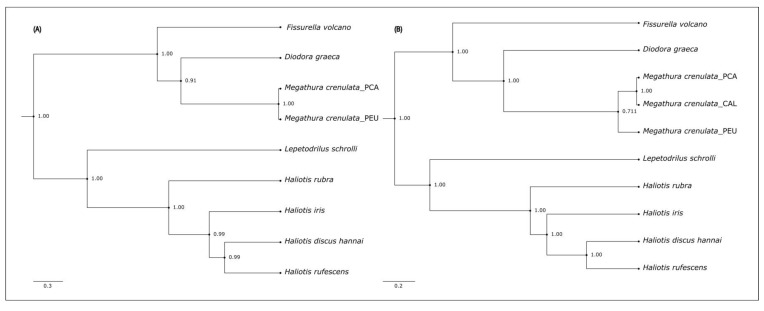
Inferred Bayesian phylogenetic relationships among eight species of gastropods. (**A**) Complete mitogenome analysis. (**B**) Analysis with three mitochondrial genes *ATP8*, *ND6*, and *COX3*. Node support values are from Bayesian bootstrap proportions.

The Bayesian phylogenetic analysis utilized the complete mitogenomes of *M. crenulata* from PCA and PEU, along with those from seven other species of marine gastropods, including two from the Fissurellidae family, four from the Haliotidae family, and one from the Lepetodriloidea family (Table 1). The complete mtDNA analysis revealed that *M. crenulata* clusters with the other Fissurellidae species (i.e., *F. volcano* and *D. graeca*) to form a monophyletic group with high node support value (>90%; Figure 2A). Similarly, *Lepetodrilus schrolli* from the Lepetodriloidea family is a sister clade to the four Haliotidae species (*Haliotis rubra*, *H. rufescens*, *H. iris,* and *H. discus hannai*) and forms a distinct monophyletic group, sister to Fissurellidae. The phylogenetic tree topology and branch lengths remain consistent when considering only the 13 PCGs of the evaluated species. In this sense, *M. crenulata* and *D. graeca* are in the same clade (100% node support), grouped with greater similarities to *F. volcano* (91% node support) compared to the other gastropods analyzed in this study. However, considering the rearrangement of the genes, *M. crenulata* and *D. graeca* share the same genes in the same order, unlike *F. volcano*, where this genome reports a lower number of tRNAs and different rearrangement (Figure S2).

The Bayesian phylogenetic analysis of only three mitochondrial genes *ATP8*, *ND6*, and *COX3* was made to include the *M. crenulata* sample from CAL, revealing similar patterns that those observed using the complete mitogenome with slight differences among node support values (Figure 2A vs. Figure 2B).

### 3.3. Genetic Diversity and Population Structure

**Figure 3:**
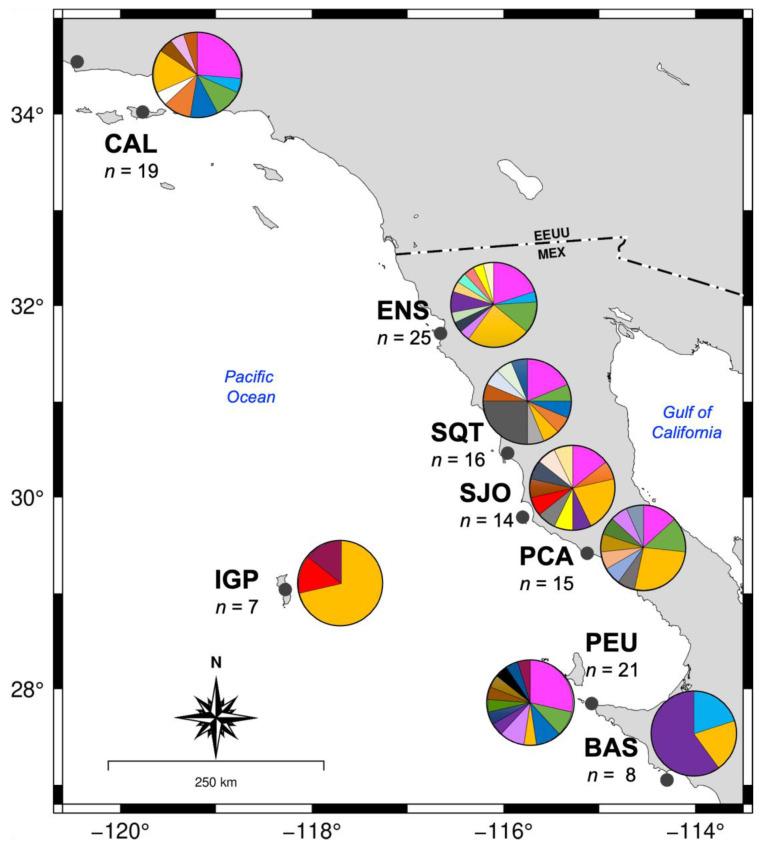
Haplotype frequency and diversity of the *16S rRNA* and *COX1* genes in eight locations of *M. crenulata*. California (CAL), Ensenada (ENS), San Quintín (SQT), Isla San Jerónimo (SJO), Puerto Canoas (PCA), Punta Eugenia (PEU), Bahía Asunción (BAS), and Isla Guadalupe (IGP). The circular diagrams indicate the diversity of haplotypes, colors indicate individual haplotypes. *n*: sample size per locality.

Two mitochondrial genes (*16S rRNA* and *COX1*) were amplified in 125 specimens of *M. crenulata* collected from eight locations along its geographical distribution. The fragments recovered were 406 bp and 747 bp long, respectively. Genetic analysis was then performed using the concatenated fragment, *16SrRNA-COX1*, which was 1153 bp overall. The global haplotypic and nucleotide diversity means were high (*h* = 0.915 and π = 0.002; Table 2). In total, 46 haplotypes were observed, and all localities had a high haplotypic diversity (*h* > 0.9), except for the localities of Bahía Asunción (*h* = 0.893 ± 0.111) and Isla Guadalupe (*h* = 0.524), which had moderate values. The nucleotide diversities by locality were similar; values ranged between 0.002 and 0.003, except for Isla Guadalupe, which had low nucleotide diversity values (0.0005, Table 2). Most localities had more than ten haplotypes, with the same two mentioned above, Bahía Asunción and Isla Guadalupe, having the fewest number of haplotypes, six and three, respectively (Table 2). Haplotype 7 (H7) was found in all the localities and was the most common haplotype, followed by haplotype 1 (H1), which was observed in six locations (CAL, ENS, SQT, SJO, PCA, and PEU). The remaining haplotypes were dispersed among all locations (Figure 3). Overall, Isla Guadalupe (IGP) presented the lowest genetic diversity in terms of haplotypic and nucleotide diversity and the total number of haplotypes.

Pairwise *φ_ST_* values ranged from −0.001 to 0.356, most comparisons had values below 0.1. However, the comparisons between BAS and three locations (SQT, PCA, and IGP) had significant values (Table 3). However, it is important to highlight that BAS and IGP have the smallest sample sizes. The global analysis of variance indicates a variation between localities of 4.09% and a global fixation coefficient value of −0.047.

### 3.4. Historic Demography

**Figure 4:**
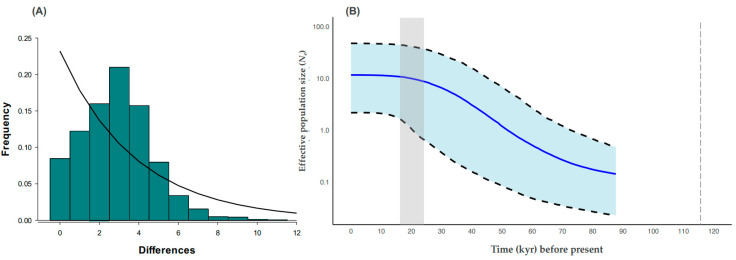
Historical demographic analyses based on *16S rRNA-COX1* concatenated haplotypes of the giant limpet *M. crenulata* along its geographical distribution. (**A**) Mismatch distribution. The bars represent the observed values, and the line represents the expected values under a constant population size model. (**B**) Bayesian skyline plot (BSP) approach. The *y*-axis is on a logarithmic scale. The *x*-axis indicates time (years) and starts at zero, corresponding to the present day. The solid blue line shows the median effective population size over time (*Ne*). The upper and bottom dashed lines represent the 95% confidence interval. The shaded grey area denotes the period during the last glacial maximum (LGM), and the vertical dashed line indicates the last interglacial (LIG) ending. A substitution rate of 0.0157 substitutions/site/million years, reported for marine invertebrates, was applied [40].

The global neutrality/demographic analyses of *Tajima’s D* (−1.943) and *Fu’s Fs* (−33.240) showed significant negative values (*p* < 0.05; Table 2). Most of *Tajima’s D* values across locations were negative, except for two that were statistically significant (ENS and IGP; Table 2). Likewise, *Fu’s Fs* values were negative, and only three were significant (SQT, SJO, and PEU). Regarding the *R*_2_ estimator, the global significant value was 0.033 (*p* = 0.002). All values per locality were low, and only in SJO, PCA, and PEU these were significant (*p* < 0.05; Table 2). Considering the concatenated mitochondrial genes *16SrRNA-COX1* among the eight locations, the observed mismatch distribution was unimodal (Figure 4A).

**Figure 5:**
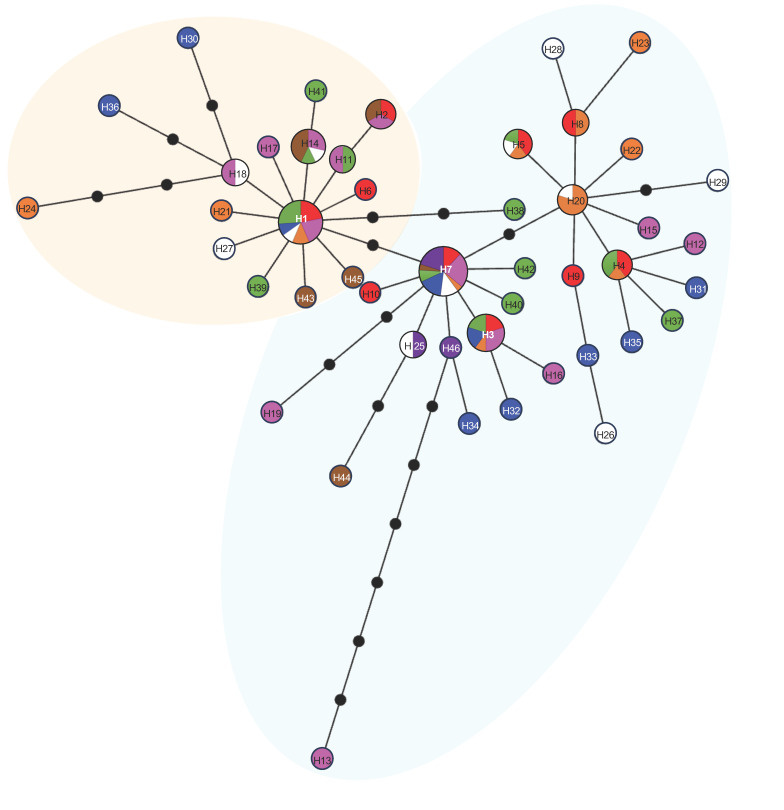
The minimum spanning network illustrates haplotypes from eight localities of *M. crenulata*. The circle sizes represent haplotype frequencies, while colors indicate the respective locality: California (CAL, red), Ensenada (ENS, pink), San Quintín (SQT, orange), Isla San Jerónimo (SJO, white), Puerto Canoas (PCA, blue), Punta Eugenia (PEU, green), Bahía Asunción (BAS, brown), and Isla Guadalupe (IGP, purple). Background circles in shaded color indicate haplogroups. Black circles represent mutational steps.

The minimum spanning network of haplotypes, considering for the eight localities, showed two distinct haplogroups, one connected to 17 haplotypes and the other to 29 haplotypes, respectively (Figure 5), and reflected a star shape from two common haplotypes, which characterizes a population expansion (Figure 5). However, out of the 46 haplotypes observed, only ten haplotypes, apart from H7, are shared among all localities. For example, H3 is shared among almost all localities except for BAS and IGP. The remaining haplotypes are distributed only among three or four localities (Figure 3), and a spatial pattern was not apparent. Finally, the Bayesian skyline plot supports a model of slow population growth of *M. crenulata* that started from approximately 80,000 years ago and that reached a stationary phase around 10,000 to 8000 years ago (Figure 4B).

## 4. Discussion

### 4.1. Mitogenome Content and Organization

This study is the first comprehensive report on the genetic organization, diversity, and structure of the mitogenome of *M. crenulata*. The mitogenome length observed in this study, ≃16,788 bp, which is consistent with the sizes of other animal mitochondrial genomes [13]. According to GenBank [16], the average length of 450 complete mitogenomes from the class Gastropoda is 15,374 bp. However, the mitogenome of *M. crenulata* is slightly shorter than those of *Fissurella volcano* (17,575 bp) and *Diodora graeca* (17,209 bp) [42], two closely related species within the Fissurellidae family. The length of the mitogenome in invertebrate species is primarily associated with the size of the control region, which can vary in size due to rearrangements of tRNAs and the presence of duplicated genes or pseudogenes [13,42].

In line with typical animal mitogenomes, consisting of a D-Loop region and 37 genes, including two ribosomal gene subunits, 13 protein-coding genes, and 18 transfer RNA genes. Regarding tRNAs, duplications of serine tRNAs (I and II) and two leucine tRNAs (I and II) were observed. The presence of duplicate tRNAs has been reported in several species of mollusks, such as *D. graeca* [18,42] and *F. volcano* [24], as well as several species of abalone such as *H. iris* [25], *H. rubra* [26], *H. rufescens* [27], *H. discus hannai* [28], *H. fulgens* [43], and *H. laevigata* [44]. This type of duplication has been seen in tRNAs related to serine, leucine, and methionine [42]. Duplication of mitochondrial tRNA genes suggests a role in the evolution and adaptation of species [14,18,42,43]. However, further studies are needed to provide insights into how tRNA gene duplications can shape the genetic landscape of mollusks and inform their evolutionary history.

These features were identified in the sequences of *M. crenulata* and *D. graeca*, with both species exhibiting the same genes and gene rearrangements, in contrast to *F. volcano* (Figure S2) [42]. The differences found in the mitogenome of *F. volcano* could be associated with its greater divergence time compared to *M. crenulata* and *D. graeca*. The relationship between species divergence times and mitochondrial gene rearrangements has been explored in other gastropods, where such rearrangements have been closely linked to phylogenetic analyses and divergence times among lineages like Caenogastropoda, Vetigastropoda, Patellogastropoda, and Heterobranchia [43].

### 4.2. Phylogenetic Analysis

*M. crenulata* is the only species within the genus *Megathura*, which resides within the subclass Vetigastropoda and order Lepetellida. The order is further divided into four superfamilies related to limpets: Haliotoidea, Lepetelloidea, Lepetodiloidea, and Fissurelloidae. Both the complete mitogenome and the 13 PCGs places of *M. crenulata* in the same clade sister of *D. graeca*, and this clade sister with *F. volcano* were analyzed; all, including *Megathura*, belonging to the Fissurelloidae superfamily. In contrast, the *Haliotis* species, from the Haliotoidea superfamily, group in a separate clade that is sister to *L. schrolli*, which belongs to the Lepetelloidea superfamily (Figure 2).

The knowledge of complete mitochondrial genomes and phylogenetic reconstructions within the Lepetellida order is still limited due to its substantial taxonomic diversity. Nevertheless, approximately 30 gastropod species have been sequenced and analyzed for phylogenetic relationships [45,46]. The arrangement observed in this study aligns with findings by other authors [18,42,47,48].

Against typical mtDNA inheritance patterns, the doubly uniparental inheritance of the mitochondrial genome has been reported in different species of mollusks, mainly within the class Bivalvia and, more frequently, in the family Mytilidae [49] in species such as *Mytilus edulis* [50,51], *Mytilus* spp. [52], and *M. galloprovincialis* [53]. This type of inheritance has also been reported in the Lepetellida mollusk *Haliotis tuberculata* [54]; however, it has been ruled out in other mollusks such as the snails *Littorina littorea*, *Nucella lapillus*, *Viviparus ater*, and including the limpet *Tectura testudinalis* [55]. Although the objectives of this study did not implicitly include a test for DUI in *M. crenulata*, our results do not show evidence for this type of inheritance; however, this pattern should be carefully evaluated in subsequent studies in the species.

### 4.3. Genetic Diversity and Population Structure

The genetic diversity patterns observed in this study for eight localities of *M. crenulata* exhibit low nucleotide diversity indices similar to those from *Concholepas concholepas*, a gastropod endemic of the Chilean coast [56] and from the pulmonated limpet *Siphonaria lessoni* from the Atlantic coast [57], both of which were evaluated through the analyses of the *COX1* gene. Likewise, when comparing it with other gastropod mollusks such as *Haliotis asinina* and *H. ovina*, we found similarities in the levels of genetic diversity associated with mitochondrial genes [58].

In general, *M. crenulata* shows moderate-to-high haplotypic diversity and low nucleotide diversity, reflecting a high number of closely related haplotypes, suggesting that the population has experienced recent population expansion along with an accumulation of mutations [59]. Populations with such characteristics are often considered metapopulations, where levels of genetic structure are low or nonexistent. This pattern has been observed in other invertebrates, such as *C. concholepas* [56].

However, the genetic structure analysis (*φ_ST_*) of *M. crenulata* revealed significant differences (*p* < 0.006) in pairwise comparisons between the following locations: BAS vs. SQT, BAS vs. PCA, IGP vs. PEU, and BAS vs. IGP. These genetic differences may be linked to larval dispersal and oceanographic currents, as noted for the pink abalone *H. corrugata* [60]. For example, Bahía Sebastián Vizcaíno oceanographically separates the coastal northern localities (CAL, ENS, SQT, SJO, and PCA) from the southern ones (PEU and BAS). From September to October, eddies are formed inside this bay [61], coinciding with the spawning season of the keyhole limpet *M. crenulata* [62]. This phenomenon, combined with a short period of larval dispersal, would avoid the north–south distribution of lecithotrophic larvae. In the specific case of genetic divergence between IGP and BAS and the low diversity values found at both sites, we must consider the biases associated with the small number of samples collected at each locality. However, despite potential sample biases in our study, other studies that have used neutral SNP markers have shown evidence of genetic divergence in samples from the same geographical region. That is, other marine snails, such as the green abalone (*H. fulgens*) and pink abalone (*H. corrugata*), found to be from IGP when compared to coastal locations along the Baja California Peninsula, have shown significant genetic differences [63,64]. Future studies are needed to explore current gene flow in *M. crenulata* and other mollusks, particularly in the more oceanic locality of IGP, to assess biogeographical patterns in this region and taxa.

### 4.4. Historic Demography

The global and significant *Tajima’s D* value of −1.943, the global and significant *Fu’s F* value of −33.24, and the low *R*_2_ (0.033), combined with a global unimodal mismatch distribution and a star-type network, all suggest recent population expansion or an excess of haplotypes with low frequency [65,66,67,68]. This finding correlates with the wide larval distribution of *M. crenulata* throughout its range [69,70,71] and supports hypotheses from previous studies [56,72].

The Bayesian skyline plot (BSP) analysis shows a model of population growth of *M. crenulata* that started before the last interglacial (LIG) era and continued after it. It is likely that the increase in temperature, changes in sea levels, and the beginning of the stabilization of environmental conditions [73,74] may have favored the adaptation of various species to the new conditions and likely benefited the processes of population expansion. Recent population stabilization is then observed for the species according to BSP. Population stabilization patterns in various mollusk species such as *Owenia fusiformis*, *Pectinaria koreni* [75], and *Tritia neritea* [76] have been documented from the onset or during the last glacial maximum (LGM) era (23–19 thousand years ago [74]). In this study, we document this pattern for *M. crenulata*, which shows a demographic stabilization that started 10,000–8000 years ago and is still ongoing. Additionally, the genetic analysis of *M. crenulata* shows a population expansion; this is similar to that reported for *Tritia neritea* [76] and for *Thaisella chocolate* [77], evaluated with *16s rRNA* and *COXI* markers.

## 5. Conclusions

In summary, the information obtained in the present work indicates that the mitochondrial genome of *M. crenulata* has a length of 16,787–16,788 bp and is composed of a total of 22 transfer RNA genes, 13 protein-coding genes (PCGs), and two ribosomal RNAs, and a D-Loop region of 461pb. Additionally, monophyletic superfamily groups were validated by our phylogenetic analyses, suggesting that these superfamilies share a common ancestor and evolved as distinct evolutionary lineages, reinforcing the current taxonomic classifications. Finally, based on the population analyses using mitochondrial markers, we found evidence of a population expansion between 80,000 and 10,000 years ago. Oceanographic barriers, such as eddies, may have influenced the genetic divergence observed in the southernmost localities by restricting larval dispersal, reducing connectivity and gene flow between these sites, but overall, we detect high genetic connectivity among localities.

Future studies should focus on the genetic diversity, connectivity, and population demography of *M. crenulata* in oceanic regions such as Isla Guadalupe (IGP) and locations further south in its range, like Bahía Asunción (BAS). These studies should include larger sample sizes and more informative molecular markers, such as neutral and adaptive SNPs, as the latter had better resolution and genome coverage to identify divergences and signals of adaptation. Given the low genetic diversity and divergence observed in the species’ southern biogeographical range, special attention should be given to the potential effects of extreme climate changes, including ocean warming. Such changes could exacerbate genetic differentiation or lead to population isolation or extinction in the near future. This study provides valuable insights into the giant keyhole limpet and helpful information for its management and conservation (for example, to establish protected areas, catching seasons, and broodstock collection areas, among others) as a critical resource for fisheries and potential aquaculture resources in the Baja California Peninsula.

## Figures and Tables

**Table 1:** List of taxa from the order Lepetellida used for the phylogenetic analyses and their GenBank accession numbers.

Superfamily	Taxon	GenBank Number	Length pb	CDS Length pb	3 CDS Length pb	Source
Fissurelloidea	*M. crenulata*—PCA	OR911353	16,788	11,294	1488	This study
Fissurelloidea	*M. crenulata*—PEU	PQ152234	16,787	11,118	1449	This study
Fissurelloidea	*M. crenulata*—CAL (*ATP8*)	PQ152231	-	-	1488	This study
Fissurelloidea	*M. crenulata*—CAL (*NAD6*)	PQ152232	-	-	1488	This study
Fissurelloidea	*M. crenulata*—CAL (*COX3*)	PQ152233	-	-	1488	This study
Fissurelloidea	*Diodora graeca*	KT207825	17,209	11,535	1498	[18]
Fissurelloidea	*Fissurella volcano*	NC_016953	17,575	11,412	1434	[24]
Haliotoidea	*Haliotis iris*	NC_031361	17,131	11,262	1458	[25]
Haliotoidea	*Haliotis rubra*	NC_005940	16,907	11,253	1455	[26]
Haliotoidea	*Haliotis rufescens*	NC_036928	16,646	11,253	1452	[27]
Haliotoidea	*Haliotis discus hannai*	EU595789	15,784	11,055	1287	[28]
Lepetodriloidea	*Lepetodrilus schrolli*	KR297250	15,579	11,406	1518	[18]

**Table 2:** Genetic diversity and demography analyses of the *16SrRNA-COX1* concatenated mitochondrial genes of *M. crenulata* were assessed at eight locations along its geographical distribution. *Tajima D* (*D_T_*), *Fu’s of F* (*Fs*), and *R*_2_ of Ramos-Onsins and Rozas, values and their statistical significance per locality.

Locality	Genetic Diversity						Neutrality Analyses					
	*n*	*H*	*S*	*h* ± S.D.	*π* ± S.D.	*θ* ± S.D.	*D_T_*	*p*-Value	*Fs*	*p*-Value	*R*_2_	*p*-Value
CAL	19	10	13	0.906 ± 0.045	0.0027 ± 0.0002	3.130 ± 1.697	−0.589	0.302	−2.792	0.101	0.105	0.190
ENS	25	13	24	0.903 ± 0.038	0.0028 ± 0.0005	3.292 ± 1.751	−1.778	**0.019**	−4.662	0.050	0.078	0.057
SQT	16	11	12	0.925 ± 0.050	0.0028 ± 0.0004	3.300 ± 1.790	−0.343	0.407	−4.763	**0.017**	0.121	0.298
SJO	14	11	14	0.956 ± 0.045	0.0030 ± 0.0004	3.570 ± 1.928	−0.775	0.242	−5.444	**0.011**	0.092	**0.027**
PCA	15	10	18	0.924 ± 0.053	0.0033 ± 0.0005	3.840 ± 1.044	−1.252	0.097	−3.115	0.078	0.081	**0.010**
PUE	21	13	16	0.914 ± 0.049	0.0027 ± 0.0003	3.179 ± 1.712	−1.054	0.145	−6.002	0.013	0.081	**0.039**
BAS	8	6	10	0.893 ± 0.111	0.0027 ± 0.0006	3.150 ± 1.821	−0.916	0.202	−1.500	0.143	0.126	0.063
IGP	7	3	2	0.524 ± 0.209	0.0005 ± 0.0002	0.571 ± 0.521	−1.237	**0.000**	−0.922	0.173	0.226	0.560
Global	125	46	48	0.915 ± 0.015	0.0028 ± 0.0001	3.004 ± 1.861	−1.944	**0.004**	−33.240	**0.000**	0.034	**0.003**

*n* = samples, *H* = haplotypes, *S* = number of segregating sites, *h* = haplotypic diversity, *π =* nucleotide diversity, *θ* = genetic distance, and *S.D.* = standard deviation. Significant probability values (*p* ≤ 0.05) are shown in bold.

**Table 3:** Comparison matrix of *φ_ST_* values and *p*-values associated to the test among eight localities of *M. crenulata*. Global *φ_ST_*: 0.047.

	CAL	ENS	SQT	SJO	PCA	PEU	BAS	IGP
**CAL**		0.279	0.495	0.855	0.693	0.603	0.018	0.045
**ENS**	0.009		0.018	0.162	0.396	0.612	0.153	0.072
**SQT**	−0.001	0.113		0.414	0.198	0.054	**0.001**	0.018
**SJO**	−0.035	0.024	−0.009		0.459	0.405	0.018	0.027
**PCA**	−0.024	0.000	0.036	−0.007		0.315	**0.001**	0.261
**PEU**	−0.015	−0.015	0.073	−0.004	0.004		0.135	**0.000**
**BAS**	0.134	0.040	0.235	0.101	0.134	0.045		**0.000**
**IGP**	0.113	0.079	0.205	0.124	0.023	0.142	0.356	

The global *p*-value was 0.011. Statistical *p*-values are displayed above the diagonal; bold values are significant after Bonferroni’s correction (*p* ≤ 0.006).

## Data Availability

The mitochondrial sequences generated in this research have been submitted to GenBank (https://www.ncbi.nlm.nih.gov/genbank/; accessed in 1 August 2024), and the accession numbers are OR911353, PQ152234, PQ152231, PQ152232, and PQ152233. Furthermore, Supplementary Materials have been included to aid reader comprehension.

## Supplementary Materials

The following supporting information can be downloaded at: https://www.mdpi.com/article/10.3390/genes15101303/s1. Figure S1. Secondary structures of four tRNAs of *M. crenulata* mitogenome. (A) *tRNA-Asparagine*, (B) *tRNA-Proline*, (C) *tRNA-Arginine*, and (D) *tRNA-Serine*. Figure S2. Comparison of the arrangement of genes in the mitochondrial genome of *F. volcano* (GenBank number: NC_016953), *M. crenulata* (GenBank number: this study), and *D. graeca* (GenBank number: KT207825) based on the published sequences. Genes in different arrangements are highlighted in colors. Table S1. Position, length, and strand direction (+/−) of the mitogenome genes of *M. crenulata*, with initiation and termination codons for protein-coding genes. Table S2. Summary of nucleotide differences from *M. crenulata* mitogenome from Puerto Canoas (PCA) and Punta Eugenia (PEU) samples. Table S3. Nucleotide differences between *M. crenulata* mitogenomes obtained from PCA and PEU samples. Regions with non-silent mutations are described.
