# Supplementary material for: Genetic Insights into the Giant Keyhole Limpet (Megathura crenulata), an Eastern Pacific Coastal Endemic: Complete Mitogenome, Phylogenetics, Phylogeography, and Historical Demography
Source: Genes (Basel). 2024 Oct 8;15(10):1303. doi: 10.3390/genes15101303 (PMC11507411; doi:10.3390/genes15101303)
Supplement: Supplementary file 1 [file genes-15-01303-s001.zip › genes-3214707-supplementary.pdf]

# Genetic insights into the giant keyhole limpet (*Megathura crenulata*) an Eastern Pacific coastal endemic: complete mitogenome, phylogenetics, phylogeography, and historical demography

Brenda Bonett-Calzada[1], Fausto Valenzuela-Quiñonez[2], Miguel A. Del Río-Portilla[1*], Natalia J. Bayona-Vásquez[3], Carmen E. Vargas-Peralta[1], John R. Hyde[4], Fabiola Lafarga-De la Cruz[1*]

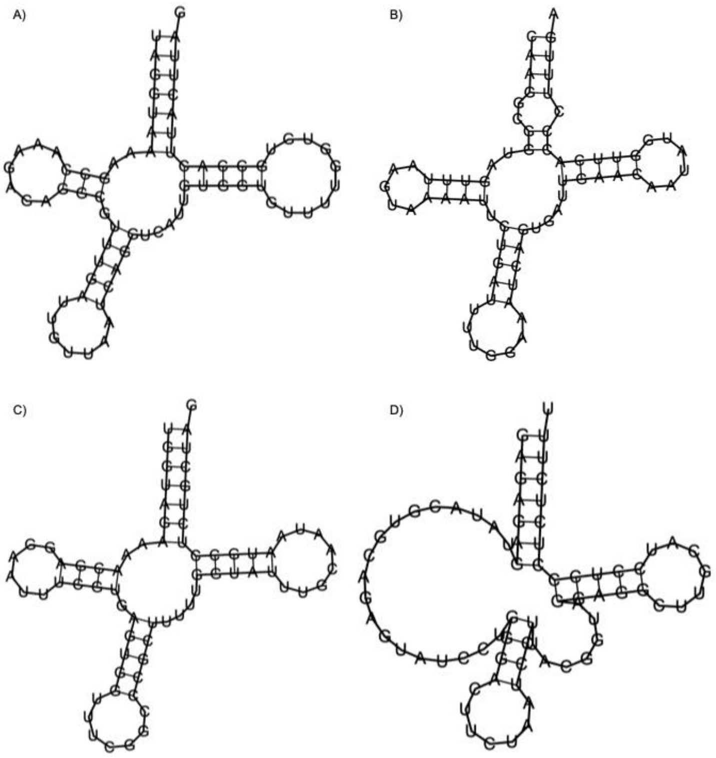


**Figure S1.** Secondary structures of four tRNAs of *M. crenulata* mitogenome A) tRNA-Asparagine, B) tRNA-Proline, C) tRNA-Arginine, and D) tRNA-Serine.

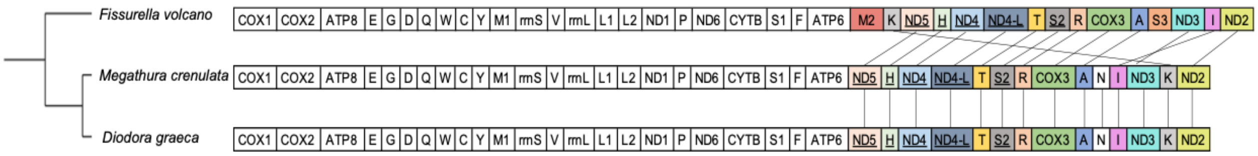


**Figure S2.** Comparison of the arrangement of genes in the mitochondrial genome of *Fissurella volcano* (GenBank number: NC_016953), *M. crenulata* (GenBank number: this study), and *Diodora graeca* (GenBank number: KT207825) based on the published sequences. Genes in different arrangements are highlighted in colors.

**Table S1**. Position, length, and strand direction (+/-) of the mitogenome genes of *M. crenulata*, with initiation and termination codons for protein-coding genes.

| Gene | Start | Stop | Length (pb) | Strand | Initiation codon | Termination codon/Anticodon | ign |
|---|---|---|---|---|---|---|---|
| COX1 | 1 | 1533 | 1533 | + | ATG | TAA | 46 |
| COX2 | 1580 | 2273 | 694 | + | ATG | TAA | 93 |
| ATP8 | 2367 | 2549 | 183 | + | ATG | TAA | 27 |
| tRNA-Glu | 2577 | 2643 | 67 | + | | TTC | 17 |
| tRNA-Gly | 2661 | 2729 | 69 | + | | TCC | 16 |
| tRNA-Asp | 2746 | 2814 | 69 | + | | GTC | 45 |
| tRNA-Gln | 2860 | 2932 | 73 | + | | TTG | 13 |
| tRNA-Trp | 2946 | 3013 | 68 | + | | TCA | 35 |
| tRNA-Cys | 3049 | 3117 | 69 | + | | GCA | 5 |
| tRNA-Tyr | 3123 | 3192 | 70 | + | | GTA | 41 |
| tRNA-Met | 3234 | 3300 | 67 | + | | CAT | 30 |
| 16s-rRNA | 3331 | 4339 | 1009 | + | | | 7 |
| tRNA-Val | 4347 | 4415 | 69 | + | | TAC | 0 |
| 12s-rRNA | 4416 | 5890 | 1475 | + | | | 0 |
| tRNA-LeuI | 5891 | 5960 | 70 | + | | TAG | 24 |
| tRNA-LeuII | 5985 | 6053 | 69 | + | | TAA | 3 |
| ND1 | 6057 | 7049 | 993 | + | ATG | TAA | 11 |
| tRNA-Pro | 7061 | 7128 | 68 | + | | | 2 |
| ND6 | 7131 | 7655 | 525 | + | ATG | TAG | 20 |
| CYTB | 7676 | 8815 | 1140 | + | ATG | TAG | 42 |
| tRNA-SerI | 8858 | 8863 | 6 | + | | | 86 |
| tRNA-Phe | 8950 | 9018 | 69 | + | | GAA | 31 |
| ATP6 | 9050 | 9745 | 696 | + | ATG | TAA | 0 |
| D-Loop | 9745 | 10207 | 461 | + | | | 0 |
| ND5 | 10207 | 11937 | 1731 | - | ATG | TAA | 0 |
| tRNA-His | 11938 | 12004 | 67 | - | | GTG | 19 |
| ND4 | 12024 | 13421 | 1398 | - | ATA | TAA | 1 |
| ND4-L | 13423 | 13719 | 297 | - | ATG | TAA | 6 |
| tRNA-Thr | 13726 | 13794 | 69 | + | | TGT | 5 |
| tRNASer-II | 13800 | 13867 | 68 | - | | TGA | 50 |
| tRNA-Arg | 13918 | 13988 | 71 | + | | | 14 |
| COX3 | 14003 | 14782 | 780 | + | ATG | TAA | 45 |
| tRNA-Ala | 14828 | 14895 | 68 | + | | TGC | 6 |
| tRNA-Asn | 14902 | 14975 | 74 | + | | | 2 |
| tRNA-Ile | 14978 | 15045 | 68 | + | | GAT | 1 |
| ND3 | 15047 | 15400 | 354 | + | ATG | TAG | 23 |
| tRNA-Lys | 15424 | 15491 | 68 | + | | TTT | 130 |
| ND2 | 15622 | 16746 | 1125 | + | ATG | TAA | |

Standard abbreviations of Protein Coding Genes (PCGs) are used. Three-letter abbreviations are listed for tRNA genes. The numbers of intergenic (ign) nucleotides are shown. Termination codon for PCGs and anticodon for tRNAs.

**Table S2.** Summary of nucleotide differences from *M. crenulata* mitogenome from Puerto Canoas (PCA) and Punta Eugenia (PEU) samples.

| Gen | Total nucleotides difference | Nucleotides with aminoacid change | Nucleotides with silent mutation |
|---|---|---|---|
| *ATP6* | 3 | 1 | 2 |
| *CYTB* | 5 | 0 | 5 |
| *COX1* | 19 | 12 | 7 |
| *COX2* | 4 | 0 | 4 |
| *COX3* | 2 | 0 | 2 |
| *rRNA* | 8 | 0 | 8 |
| *ND1* | 3 | 1 | 2 |
| *ND2* | 3 | 0 | 3 |
| *ND3* | 1 | 0 | 1 |
| *ND4* | 3 | 0 | 3 |
| *ND5* | 13 | 1 | 12 |
| *ND6* | 2 | 0 | 2 |
| *tRNA-LeuII* | 1 | 0 | 1 |
| *tRNA-Phe* | 1 | 0 | 1 |
| Control region | 16 | 0 | 16 |
| Intergenes | 5 | 0 | 5 |
| Total | 89 | 15 | 74 |

**Table S3.** Nucleotide differences between *M. crenulata* mitogenomes obtained from PCA and PEU samples. Regions with non-silent mutations are described.

| Position in sequence | *M. crenulata* PCA | *M. crenulata* PEU | Gene | Aminoacid change |
|---|---|---|---|---|
| 424-426 | T | A | *COX1* | Leucine -> Serine |
| | T | G | *COX1* | |
| | G | A | *COX1* | |
| 427 - 429 | G | T | *COX1* | Alanine -> Serine |
| | T | G | *COX1* | |
| | A | G | *COX1* | |
| 430-432 | T | A | *COX1* | Isolueucine -> Glutamate |
| | T | A | *COX1* | |
| | T | G | *COX1* | |
| 433-435 | T | A | *COX1* | Phenylalanine -> Glutamate |
| | T | G | *COX1* | |
| 436 | T | C | *COX1* | Serine -> Proline |
| 6304 | G | A | *ND1* | Valine -> Isoluecine |
| 9057 | G | A | *ATP6* | Glycine -> Serine |
| 10227 | A | G | *ND5* | Alanine -> Leucine |
